# Correction: Integrated culturing, modeling and transcriptomics uncovers complex interactions and emergent behavior in a three-species synthetic gut community

**DOI:** 10.7554/eLife.53217

**Published:** 2019-11-04

**Authors:** Kevin D'hoe, Stefan Vet, Karoline Faust, Frédéric Moens, Gwen Falony, Didier Gonze, Verónica Lloréns-Rico, Lendert Gelens, Jan Danckaert, Luc De Vuyst, Jeroen Raes

D'hoe K, Vet S, Faust K, Moens F, Falony G, Gonze D, Lloréns-Rico V, Gelens L, Danckaert J, Vuyst LD, Raes J. 2018. Integrated culturing, modeling and transcriptomics uncovers complex interactions and emergent behavior in a three- species synthetic gut community. *eLife* **7**:e37090. doi: 10.7554/eLife.37090.

Published 16, October 2018

We noticed a few minor errors after publication.

First, in Figure 6, bacterial abundance in panels D, E and F was given in mM instead of counts per mL. The caption did indicate the correct unit, but to avoid confusion, we have now corrected the units given in the panels.

The corrected Figure 6 is shown here:

**Figure:**
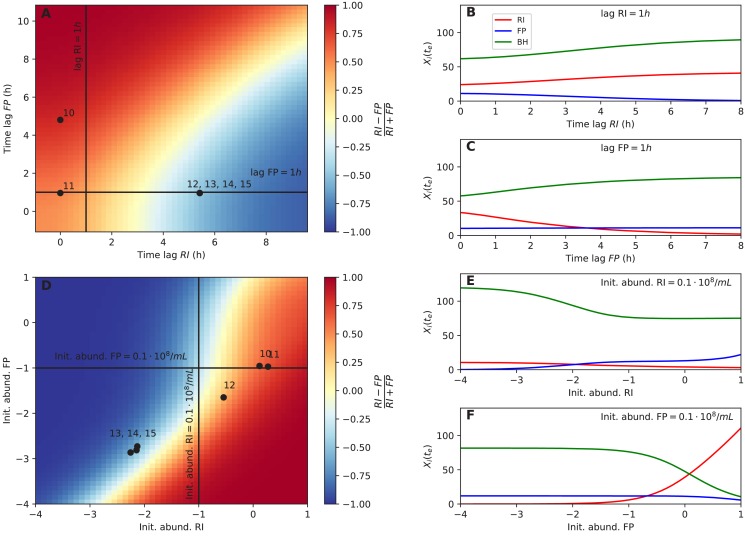


The originally published Figure 6 is shown for reference:

**Figure:**
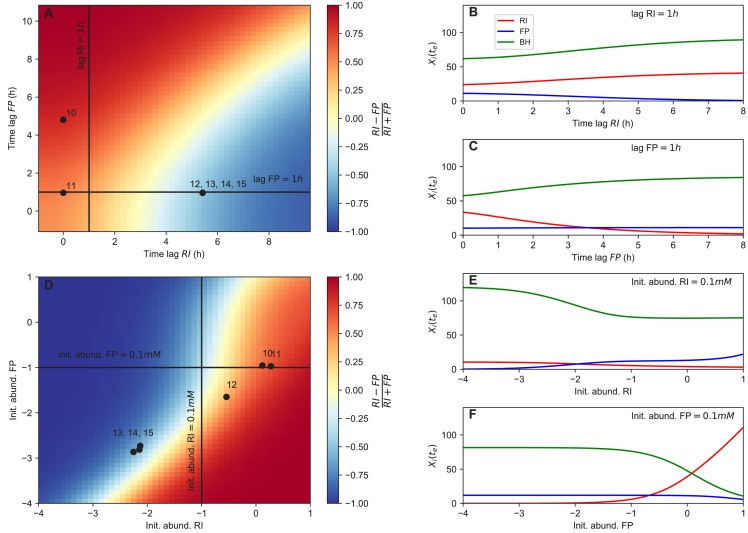


Second, we made a mistake in the set of equations describing the changes of metabolite concentrations in the Model definition section of the Methods part and omitted equations for metabolite-specific growth functions that should have been there. However, the model was simulated with the correct equations (see model source code: https://doi.org/10.7554/eLife.37090.028).

The corrected equations for the changes of metabolite concentrations and the previously missing equations for metabolite-specific growth are given here:

$$\begin{array}{ll} \frac{dS_{fructose}}{dt} & =-\nu_{RI,fructose}\Phi_{RI}X_{RI}-\nu_{FP,fructose}\Phi_{FP}X_{FP}-\nu_{BH,fructose}\Phi_{BH,fructose}X_{BH}\\ \frac{dS_{formate}}{dt} & =\alpha_{RI,formate}\Phi_{RI}X_{RI}+\alpha_{FP,formate}\Phi_{FP}X_{FP}-\nu_{BH,formate}\Phi_{BH,formate}X_{BH}\\ \frac{dS_{acetate}}{dt} & =-\nu_{RI,acetate}\Phi_{RI,acetate}X_{RI}-\nu_{FP,acetate}\Phi_{FP,acetate}X_{FP}+\alpha_{BH,acetate}\Phi_{BH}X_{BH}\\ \frac{dS_{butyrate}}{dt} & =\alpha_{RI,butyrate}\Phi_{RI}X_{RI}+\alpha_{FP,butyrate}\Phi_{FP}X_{FP}\\ \frac{dS_{unknown}}{dt} & =-\nu_{FP,unknown}\Phi_{FP}X_{FP}\\ \frac{dS_{H_{2}}}{dt} & =\alpha_{RI,H_{2}}\Phi_{RI}X_{RI}+\alpha_{RI,H_{2}}\Phi_{BH}X_{BH}\\ \frac{dS_{CO_{2}}}{dt} & =\alpha_{RI,CO_{2}}\Phi_{RI}X_{RI}+\alpha_{FP,CO_{2}}\Phi_{FP}X_{FP}+\alpha_{BH,CO_{2}}\Phi_{BH}X_{BH} \end{array}$$

$$\begin{array}{ll} \Phi_{RI,acetate} & =\Gamma_{RI}(Q_{RI})\mu_{RI}w_{RI}\frac{S_{fructose}}{K_{RI,fructose}+S_{fructose}}\frac{S_{acetate}}{K_{RI,acetate}+S_{acetate}}\\ \Phi_{FP,acetate} & =\Gamma_{FP}(Q_{FP})\mu_{FP}w_{FP}\frac{S_{unknown}}{K_{FP,unknown}+S_{unknown}}\frac{S_{acetate}}{K_{FP,fructose}+S_{fructose}}\frac{S_{acetate}}{K_{FP,acetate}+S_{acetate}}\\ \Phi_{BH,fructose} & =\Gamma_{BH}(Q_{BH})\mu_{BH}\frac{S_{fructose}}{K_{BH,fructose}+S_{fructose}}\\ \Phi_{BH,fructose} & =\Gamma_{BH}(Q_{BH})\mu_{BH}w_{BH}\frac{S_{formate}}{K_{BH,formate}+S_{formate}} \end{array}$$

The subscripts in the originally published equations for the changes in metabolite concentrations were incorrect, as the subscripts referred to the complete growth functions instead of the metabolite-specific growth functions. The originally published equations are shown here for reference:

$$\begin{array}{ll} \frac{dS_{fructose}}{dt} & =-\nu_{RI,fructose}\Phi_{RI}X_{RI}-\nu_{FP,fructose}\Phi_{FP}X_{FP}-\nu_{BH,fructose}\Phi_{BH}X_{BH}\\ \frac{dS_{formate}}{dt} & =\alpha_{RI,formate}\Phi_{RI}X_{RI}+\alpha_{FP,formate}\Phi_{FP}X_{FP}-\nu_{BH,formate}\Phi_{BH}X_{BH}\\ \frac{dS_{acetate}}{dt} & =-\nu_{RI,acetate}\Phi_{RI}X_{RI}-\nu_{FP,acetate}\Phi_{FP}X_{FP}+\alpha_{BH,acetate}\Phi_{BH}X_{BH}\\ \frac{dS_{butyrate}}{dt} & =\alpha_{RI,butyrate}\Phi_{RI}X_{RI}+\alpha_{FP,butyrate}\Phi_{FP}X_{FP}\\ \frac{dS_{unknown}}{dt} & =-\nu_{FP,unknown}\Phi_{FP}X_{FP}\\ \frac{dS_{H_{2}}}{dt} & =\alpha_{RI,H_{2}}\Phi_{RI}X_{RI}+\alpha_{RI,H_{2}}\Phi_{BH}X_{BH}\\ \frac{dS_{CO_{2}}}{dt} & =\alpha_{RI,CO_{2}}\Phi_{RI}X_{RI}+\alpha_{FP,CO_{2}}\Phi_{FP}X_{FP}+\alpha_{BH,CO_{2}}\Phi_{BH}X_{BH} \end{array}$$

Finally, there is also a mistake in the legend of Supplementary File 2. Instead of defining the production and consumption rate parameters, the production rate parameter was listed twice.

The corrected part of the legend is:

α: production rate in mM/(10ˆ8 bacterial counts/mL)

ν: consumption rate in mM/(10ˆ8 bacterial counts/mL)

The originally published part of the legend is:

α: production rate in mM/(10ˆ8 bacterial counts/mL)

α: production rate in mM/(10ˆ8 bacterial counts/mL)

We apologize for these errors. To the best of our knowledge, everything in the corrected article is accurate as presented.

The article has been corrected accordingly.

