# Supplementary material for: Integrated culturing, modeling and transcriptomics uncovers complex interactions and emergent behavior in a three-species synthetic gut community
Source: eLife. 2018 Oct 16;7:e37090. doi: 10.7554/eLife.37090 (PMC6237439; doi:10.7554/eLife.37090)
Supplement: Transparent reporting form [file elife-37090-transrepform.docx]

***eLife’s* transparent reporting form**

We encourage authors to provide detailed information *within their submission* to facilitate the interpretation and replication of experiments. Authors can upload supporting documentation to indicate the use of appropriate reporting guidelines for health-related research (see [EQUATOR Network](http://www.equator-network.org/%20)), life science research (see the [BioSharing Information Resource](https://biosharing.org/)), or the [ARRIVE guidelines](http://www.plosbiology.org/article/info:doi/10.1371/journal.pbio.1000412) for reporting work involving animal research. Where applicable, authors should refer to any relevant reporting standards documents in this form.

If you have any questions, please consult our Journal Policies and/or contact us: [editorial@elifesciences.org](mailto:editorial@elifesciences.org).

**Sample-size estimation**

- You should state whether an appropriate sample size was computed when the study was being designed
- You should state the statistical method of sample size computation and any required assumptions
- If no explicit power analysis was used, you should describe how you decided what sample (replicate) size (number) to use

Please outline where this information can be found within the submission (e.g., sections or figure legends), or explain why this information doesn’t apply to your submission:

In total, we performed 25 fermentation experiments in well-controlled conditions (an overview is provided in Table 1) and developed a model to describe these 25 time series. We derive our main insights from the goodness of fit of two different model parameterizations. Thus, we do not compare two sets of biological replicates, but instead compare two model parameterizations, each of which describing 25 experiments belonging to 9 different sets of biological replicates. In each parameterization, the model is fitted on a sub-set of 5 experiments, leaving 20 experiments for model validation and 12 co-culture experiments for comparison. We consider this to be sufficient for our question and did not formally compute statistical power.

**Replicates**

- You should report how often each experiment was performed
- You should include a definition of biological versus technical replication
- The data obtained should be provided and sufficient information should be provided to indicate the number of independent biological and/or technical replicates
- If you encountered any outliers, you should describe how these were handled
- Criteria for exclusion/inclusion of data should be clearly stated
- High-throughput sequence data should be uploaded before submission, with a private link for reviewers provided (these are available from both GEO and ArrayExpress)

Please outline where this information can be found within the submission (e.g., sections or figure legends), or explain why this information doesn’t apply to your submission:

Most experiments were performed in at least two biological replicates, with three technical replicates for each data point. A biological replicate refers to a full run of a fermenter for a particular mono- or co-culture. Technical replicates refer to samples taken in triplicate at each time point in a particular fermentation experiment. No treatment of outliers was applied. Fermentation data have been submitted to Dryad and sequencing data to SRA. Private links for reviewers are provided in the submission.

**Statistical reporting**

- Statistical analysis methods should be described and justified
- Raw data should be presented in figures whenever informative to do so (typically when N per group is less than 10)
- For each experiment, you should identify the statistical tests used, exact values of N, definitions of center, methods of multiple test correction, and dispersion and precision measures (e.g., mean, median, SD, SEM, confidence intervals; and, for the major substantive results, a measure of effect size (e.g., Pearson's r, Cohen's d)
- Report exact p-values wherever possible alongside the summary statistics and 95% confidence intervals. These should be reported for all key questions and not only when the p-value is less than 0.05.

Please outline where this information can be found within the submission (e.g., sections or figure legends), or explain why this information doesn’t apply to your submission:

The raw data (metabolite concentrations and species abundances) are visualized in Figure 3 of the submission, with the standard deviation across biological replicates indicated as whiskers. In addition, the mean and standard deviation of metabolite concentrations across biological replicates is provided in Table 1, which also lists the number of experiments performed. Standard deviation across technical replicates is visualized in Figures 4 and 5 with whiskers.

The goodness of model fit is computed with the root mean square error (RMSE). The RMSE is summarized across biological replicates with mean and standard deviation given in Table 1.

The unpaired two-sided Wilcoxon test was applied to test for significantly different maximal abundances in mono- and co-cultures. Corresponding p-values and confidence intervals are reported in Results (section: Comparison of mono- and co-culture data suggests ecological interactions).
The individual results for all the replicates in the RNA-seq species detection are reported in Supplementary Figure 3.

Significance of differential gene expression between mono- and tri-culture across all time points was computed with the Wald test implemented in DESeq2. Differentially expressed genes with Benjamini-Hochberg corrected p-values below 0.05 are reported in Supplementary Table 3. The code that has been used for the analysis is available at <https://github.com/vllorens/syntheticGutCommunity> and can be used to reproduce all the statistics and p-values, also for the non-differentially expressed genes.

(For large datasets, or papers with a very large number of statistical tests, you may upload a single table file with tests, Ns, etc., with reference to sections in the manuscript.)

**Group allocation**

- Indicate how samples were allocated into experimental groups (in the case of clinical studies, please specify allocation to treatment method); if randomization was used, please also state if restricted randomization was applied
- Indicate if masking was used during group allocation, data collection and/or data analysis

Please outline where this information can be found within the submission (e.g., sections or figure legends), or explain why this information doesn’t apply to your submission:

Group allocation does not apply to the current study.

**Additional data files (“source data”)**

- We encourage you to upload relevant additional data files, such as numerical data that are represented as a graph in a figure, or as a summary table
- Where provided, these should be in the most useful format, and they can be uploaded as “Source data” files linked to a main figure or table
- Include model definition files including the full list of parameters used
- Include code used for data analysis (e.g., R, MatLab)
- Avoid stating that data files are “available upon request”

Please indicate the figures or tables for which source data files have been provided:

The fermentation data (data points in Figure 3, 4 and 5) have been submitted to Dryad. The model equations are given in Material and Methods in section Model definition. Model parameters and initial values are provided in Supplementary Table 2. The model is specified in executable form in a Source Code File.

The R code for the RNA-seq related data analysis and figure generation, as well as the starting files for these analyses (i.e. output from the RNA-seq preprocessing) can be found in the following GitHub repository: https://github.com/vllorens/syntheticGutCommunity
